# The Antimicrobial Activity and Characterization of Bioactive Compounds in *Peganum harmala* L. Based on HPLC and HS-SPME-GC-MS

**DOI:** 10.3389/fmicb.2022.916371

**Published:** 2022-07-19

**Authors:** Ningning Wang, Junxia An, Zhijun Zhang, Yingqian Liu, Jianguo Fang, Zhigang Yang

**Affiliations:** ^1^School of Pharmacy, Lanzhou University, Lanzhou, China; ^2^Collaborative Innovation Center for Northwestern Chinese Medicine, Lanzhou University, Lanzhou, China; ^3^School of Chemistry and Chemical Engineering, Lanzhou University, Lanzhou, China

**Keywords:** alkaloids, HS-SPME-GC-MS, volatile organic compounds, antimicrobial activity, *Peganum harmala* L

## Abstract

*Peganum harmala* L. is a perennial herb of the Tribulus family and its aerial parts and seeds can be used as medicine in the traditional medicine of China. However, the differences in chemical components and antibacterial activity between different parts have not been reported. In this study, the chemical composition of the different parts of *P. harmala* was characterized by high-performance liquid chromatography (HPLC) and headspace-solid phase microextraction-gas chromatography-mass spectrometry (HS-SPME-GC-MS). The antimicrobial activities of the different parts and some isolated components were also carried out on 12 bacterial strains and phytopathogenic fungi. The HPLC results revealed that the contents of harmine and harmaline in the seeds were higher than that in the aerial parts. A total of 94 volatile organic compounds (VOCs) were tentatively identified by HS-SPME-GC-MS for the first time. The major components were methyl hexadecanoate, p-xylene, octane, (Z)-9-octadecanoate, ethylbenzene, methyl octadecanoate, ethyl hexadecanoate, and methyl tetradecanoate. At the concentration of 800 μg·mL^−1^, the methanol extracts of seeds showed stronger antimicrobial activities with a wide antimicrobial spectrum, inhibiting *Escherichia coli* (ATCC 24433), *Xanthomonas oryzae* (ACCC 11602), and *Xanthomonas axonopodis* with inhibitory rates of more than 90%. Furthermore, harmine and harmaline showed better antibacterial activities against all the bacteria. These findings indicated that alkaloids from *P. harmala* could account for antimicrobial activity, which could be used as lead molecules in the development of new antimicrobial drugs.

## Introduction

*Peganum harmala* L. is widely distributed in the arid areas of Northwest China, and the aerial parts and seeds of *P. harmala* can be used as medicine in the traditional medicine of Uygur, Kazak, Mongolian, and Tibetan, which possesses neuroprotective, anticancer, antiviral, larvicidal, hepatoprotective activity, antioxidant and anti-inflammatory, antitussive, expectorant, and bronchodilating effects (Li et al., 2017). It has been reported that the main compounds of *P. harmala* were alkaloids, such as β-carboline and quinazolone alkaloids and their derivatives (Jalali et al., 2021). However, the differences in chemical components between different parts have not been reported. High-performance liquid chromatography (HPLC) has the characteristics of high sensitivity, high separation efficiency, easy operation, and good repeatability (Sahu et al., 2018; Martinez-Navarro et al., 2021). Gas chromatography-mass spectrometry (GC-MS) combined with solid-phase microextraction (SPME) provides a more environmentally friendly, simple, and accurate method for the study of volatile components, which are widely used in the detection of volatile organic compounds (VOCs) in plants (Feng et al., 2022). The crushed leaves and stems of *P. harmala* have a disagreeable odor (Xu et al., 2008). However, the VOCs of *P. harmala* has not been reported using HS-SPME-GC-MS, and its volatile components in the aerial parts are still poorly understood. To explore the chemical constituents of *P. harmala* L., its aerial parts and seeds were analyzed by HPLC and HS-SPME-GC-MS.

Natural products derived from plants have significant efficacy with antibacterial activity, particularly against pathogens that could be used to overcome drug resistance (Ye et al., 2020). For example, it has been demonstrated that *P. harmala* has antimicrobial activities, effectively inhibiting the growth of *Staphylococcus aureus* (DSM 25693), *Bacillus cereus* (DSM 4313), *Bacillus cereus* (DSM4384), *Escherichia coli* (DMS 857), and *Pseudomonas aeruginosa* (ATCC 50071) (Azzazy et al., 2021). But *P. harmala* has not been reported for potential antifungal activities against phytopathogenic fungi.

In the present study, to clarify the chemicals of different parts of *P. harmala*, the HPLC method was established for the simultaneous determination of harmine, harmaline, vasicine, and vasicinone in its aerial parts and seeds. HS-SPME-GC-MS technology was also used to determine the differences of VOCs in different parts of *P. harmala*. Furthermore, extracts from different parts of *P. harmala* were assayed against 12 bacterial strains and phytopathogenic fungi. To our knowledge, this is the first time to report the HPLC, HS-SPME-GC-MS, and antimicrobial activity results of different parts of *P. harmala*.

## Materials and Methods

### Materials and Reagents

Fresh aerial parts of *P. harmala* L. were collected and dried at room temperature in October 2020 from Pakistan and Gansu of China. Dried seeds were collected from Xinjiang of China. The plants were identified by Dr. Zhigang Yang, and the voucher specimen (No. 202010002) was stored in the School of Pharmacy at Lanzhou University.

Ethanol, methanol, ethyl acetate, dichloromethane, and chloroform were purchased from Tianjin Jiatong Chemical reagent Co., Ltd., Dimethylsulfoxide (DMSO) was obtained from Sigma-Aldrich (USA); Acetonitrile was purchased from Merck (Germany).

### Extraction and Separation

The seeds of *P. harmala* (9.0 kg) were extracted under reflux with 70% ethanol for three times. The extracts (1.6 kg) were suspended in 5% HCl, then the acidic mixture was successively extracted with dichloromethane and the aqueous layer was alkalinized to pH 9 with NaOH solution, followed by exhaustive extraction with dichloromethane to obtain crude alkaloids (273 g). The crude alkaloids were subjected to silica gel column chromatography, eluting with CH_2_Cl_2_:MeOH (100:0-0:100), to obtain nine fractions (Fr.A-I) (Wang et al., 2017). Fr.E was separated on Al_2_O_3_ chromatography and eluted by chloroform and methanol to obtain compound **1** (1.4 g). Fr.F was separated on silica gel chromatography, eluting with CH_2_Cl_2_:MeOH, and obtained compound **2** (0.1 g) by recrystallization in MeOH. Compound **3** (1.4 g) was obtained from Fr.B *via* recrystallization in MeOH. Fr.B was separated on silica gel chromatography, eluting with dichloromethane and acetone, and obtained compound **4** (0.1 g). Their structures were elucidated based on nuclear magnetic resonance (NMR). The NMR experiments were performed on a Bruker AVANCE AV III-400 instrument (Bruker, Switzerland, 100 MHz for ^13^C and 400 MHz for ^1^H) with tetramethylsilane (TMS) as an internal reference.

### HPLC Chromatographic Conditions

#### Preparation of Standard Compound Solutions

The vasicine (3.53 mg), vasicinone (1.54 mg), harmine (6.66 mg), and harmaline (5.90 mg) were dissolved in methanol and configured at different gradient concentrations.

#### Preparation of Samples Solutions

In total, 0.25 g powders of different parts from *P. harmala* were extracted by ultrasonic treatment with 25 ml methanol for 30 min at 30°C using an ultrasonic cleaner (Model KQ5200E, Kunshan ultrasonic instrument Co. LTD, China). Then, the sample was filtered by a membrane filter (0.22 μm) for HPLC analysis.

#### HPLC Analysis

EasySep-3030 liquid chromatography (Shanghai Tongwei Company) included an EasySep3030 UV detector, AS-2000 automatic sampler, and EasySep3030 binary pump. The column was C18 (4.6 × 250 mm, 5 μm, Shanghai Tongwei), and the mobile phase A was acetonitrile and mobile phase B was 0.6% glacial acetic acid solution containing 0.088 mol/L ammonium acetate. The HPLC elution conditions were operated as follows: 0–7 min, 10% A; 7.1–25 min, 19% A; 25.1–37 min, 27% A; 37.1–45 min, 27–40% A; and 45.1–60 min, 100–10% A. The injection volume was 10 μl and eluted at 1.0 ml/min, the column temperature was kept at 30°C, and the detection wavelength was 265 nm (Wen et al., 2012).

### HS-SPME-GC-MS Conditions

#### Isolation and Concentration of GC-MS

In total, 1 g powder of samples was transferred immediately to a 20-ml head-space vial (Agilent, Palo Alto, CA, USA), containing NaCl saturated solution. The vials were sealed using crimp-top caps with TFE-silicone headspace septa (Agilent). At the time of SPME analysis, each vial was placed at 100°C for 5 min, then a 120-μm divinylbenzene/carboxen/polydimethylsilioxane fiber (Agilent) was exposed to the headspace of the sample for 15 min at 250°C.

#### GC-MS Conditions

After sampling, desorption of the VOCs from the fiber coating was carried out in the injection port of the GC-MS (Agilent; Model 8890, 5977B) at 250°C for 5 min in the splitless mode. The GC was equipped with a 30 m × 0.25 mm × 0.25 μm DB-5MS (5% phenyl-polymethylsiloxane) capillary column. Helium was used as the carrier gas at a linear velocity of 1.2 ml/min. The injector temperature was kept at 250°C and the detector at 280°C. The oven temperature was programmed from 40°C (3.5 min), increasing at 10°C/min to 100°C, at 7°C/min to 180°C, and at 25°C/min to 280°C, and held for 5 min. Mass spectra were recorded in electron impact (EI) ionization mode at 70 eV, using the full scan mode. The quadrupole mass detector, ion source, and transfer line temperatures were set at 150, 230, and 280°C, respectively. Mass spectra were scanned in the range *m/z* 50 to 500 amu at 1 s intervals. Identification of volatile compounds was achieved by comparing the mass spectra with the data system library (NIST) and linear retention index. The concentration of each compound in the *P. harmala* was calculated by the internal standard method.

### Determination of Antibacterial Activity

The petroleum ether and methanol extracts were prepared as follows: 0.50 g powder of seeds and the aerial parts were refluxed with 100 ml petroleum ether and methanol three times. After filtration, concentration, and evaporation, the petroleum ether and methanol extract were dried and then obtained for antibacterial activity tests. *E. coli* (ATCC 25922), *Staphylococcus aureus* (Newman), *X. oryzae* (ACCC 11602), *X. axonopodis*, and *Pectobacterium atroseptica* (ACCC 19901) were provided by the Institute of Plant Protection, Gansu Academy of Agricultural Science. The strains were removed and underlined on NB solid medium and incubated at 28°C until single colonies grew. Colonies on solid medium were picked for agricultural bacterial NB liquid medium at 28°C and 180 rpm and shaken to grow. Strains in the log growth phase were diluted to about 10^6^ CFU/ml. The samples were separately dissolved in dimethyl sulfoxide (DMSO) and then added to the liquid medium with a concentration of 1,600 μg/ml (0.5% DMSO). Before inoculating each well with 50 μl of bacterial suspension, a 2-fold series of NB dilutions of 50 μl of extracts and alkaloids were prepared in a 96-well plate (final concentrations of extracts were 800, 400, 200, 100, and 50 μg/ml; final concentrations of alkaloids were 100, 50, 25, and 12.5 μg/ml). A 100-μl suspension of the same concentration containing the same volume of DMSO was used as a control. The plates were then incubated at 28°C for 24 to 48 h and the optical density at 600 nm (OD_600_) was measured. The OD_600_ of 100 μl of NB solution containing the same concentration of DMSO or compounds was measured for correction. The inhibition rate was calculated using the following formula (Ferreira-Santos et al., 2021):


(1) $$\text{The}\;\text{corrected}\;{\text{values = OD}}_{\text{bacterial wilt}}{\text{- OD}}_{\text{no bacterial}\;\text{wilt}}$$

(2) The inhibition rate = (C-T)/C × 100%

Where C is the corrected turbidity values of control and T is the corrected turbidity values of bacterial growth treated with compounds. The MIC values were defined as the concentration of the compound that inhibited bacterial growth by, at least, 90% (the MIC_90_).

### Determination of Antifungal Activity

Seven pathogenic fungi, *Candida albicans* (ATCC 24433), *Rhizoctonia solani, Sclerotinia sclerotiorum, Botrytis cinerea, Fusarium graminearum, Magnaporthe oryzae*, and *Phytophthora capsici* were obtained from the Institute of Plant Protection, Gansu Academy of Agricultural Science. The fungi were cultured in potato dextrose agar medium (PDA) at 25°C for 3–6 days to obtain new mycelia for the antifungal assay. The mycelium growth inhibition method was used to evaluate the antifungal activity of *P. harmala*. First, the petroleum ether, methanol extracts, and alkaloids were dissolved in DMSO and then added to the PDA medium to obtain sterile media of different concentrations. Then, a 5-mm agar plug of each fungal strain was obtained from a 3-day-old PDA culture, which was inoculated in the middle of PDA plates containing samples. The same amount of DMSO (0.5%) was added to the sterile medium as a blank control. Petri dishes were incubated at 25°C, and the mycelial growth diameter was measured using the cross-bracketing method until the fungal growth of the blank control had completely covered the Petri dishes. The inhibitory rates were calculated according to the following formula (Yan et al., 2021):


(3) $$\begin{array}{l} \text{Mycelial growth inhibition rate}\;(\%)=[(C-T)/(C-5mm)]\\ \times \;100 \end{array}$$

Where C and T are the average colony diameters of the mycelium of the blank control and treatment, respectively.

### Statistical Analysis

All test data were calculated for the means and SDs using the SPSS 22.0 software, one-way ANOVA was employed for the significant differences test, and the means were compared by Duncan's tests at *P* < 0.05 and *P* < 0.01.

## Results and Discussion

### NMR Data of the Isolated Alkaloids

Compound **1** was obtained as pale yellow prismatic crystallization and showed purple fluorescence at 365 nm. ^1^H-NMR (400 MHz DMSO-*d*_6_) δppm: 11.45 (1H, s, NH), 8.17 (1H, d, *J* =5.3 Hz), 8.06 (1H, d, *J* =8.6 Hz), 7.82 (1H, d, *J* =5.3 Hz), 7.03 (1H, d, *J* =2.0 Hz), 6.87 (1H, dd, *J* =8.6, 2.0 Hz), 3.88 (3H, s, OCH_3_), and 2.74 (3H, s, CH_3_); ^13^C-NMR (100 MHz DMSO-*d*_6_) δppm: 142.4 (C-1), 138.1 (C-3), 112.4 (C-4), 123.1 (C-5), 109.5 (C-6), 160.6 (C-7), 95.1 (C-8), 135.0 (C-10), 127.7 (C-11), 115.3 (C-12), 141.7 (C-13), 55.8 (OCH_3_), and 20.8 (CH_3_). Thus, the structure of compound **1** was determined as harmaline by comparing the NMR data with the literature (Benarous et al., 2015).

Compound **2** was obtained as yellow sheet crystallization and showed bright blue fluorescence at 365 nm. ^1^H-NMR (400 MHz DMSO-*d*_6_) δppm: 11.16 (1H, s, NH), 7.43 (1H, d, *J* = 8.7 Hz), 6.86 (1H, d, *J* = 1.8 Hz), 6.71 (1H, dd, *J* = 8.7, 2.2 Hz), 3.79 (3H, s, OCH_3_), 3.65 (2H, t, *J* = 8.8 Hz), 2.70 (3H, t, 8.4 Hz), and 2.25 (3H, s, CH_3_); ^13^C-NMR (100 MHz DMSO-*d*_6_) δppm: 157.7 (C-1), 48.0 (C-3), 19.6 (C-4), 119.9 (C-5), 110.7 (C-6), 157.3 (C-7), 95.1 (C-8), 129.0 (C-10), 120.8 (C-11), 115.2 (C-12), 138.1 (C-13), 55.6 (OCH_3_), and 22.4 (CH_3_). Therefore, the structure of compound **2** was elucidated as harmine by comparing the NMR data with the literature (Mukhtar et al., 2002).

Compound **3** was obtained as white amorphous powder. ^1^H-NMR (400 MHz CDCl_3_) δppm: 11.42 (1H, s, NH), 8.16 (1H, d, *J* = 5.2 Hz), 7.80 (1H, d, *J* = 5.2 Hz), 8.05 (1H, d, *J* = 8.8 Hz), 6.84 (1H, dd, *J* = 8.8, 2.0 Hz), 7.01 (1H, d, *J* = 2.0 Hz), 3.87 (3H, s, –OCH_3_), and 2.72 (3H, s, –CH_3_); ^13^C-NMR (100 MHz CdCl_3_) δ ppm: 160.0 (C-1), 137.7 (C-3), 111.9 (C-4), 122.6 (C-5), 109.0 (C-6), 141.9 (C-7), 94.6(C-8), 141.2 (C-10), 127.2 (C-11), 114.8 (C-12), 134.5 (C-13), 55.3 (OCH_3_), and 20.3 (CH_3_). Therefore, the structure of compound **3** was determined as vasicine by comparing the NMR data with the literature (Wang et al., 2015).

**Figure 1:**
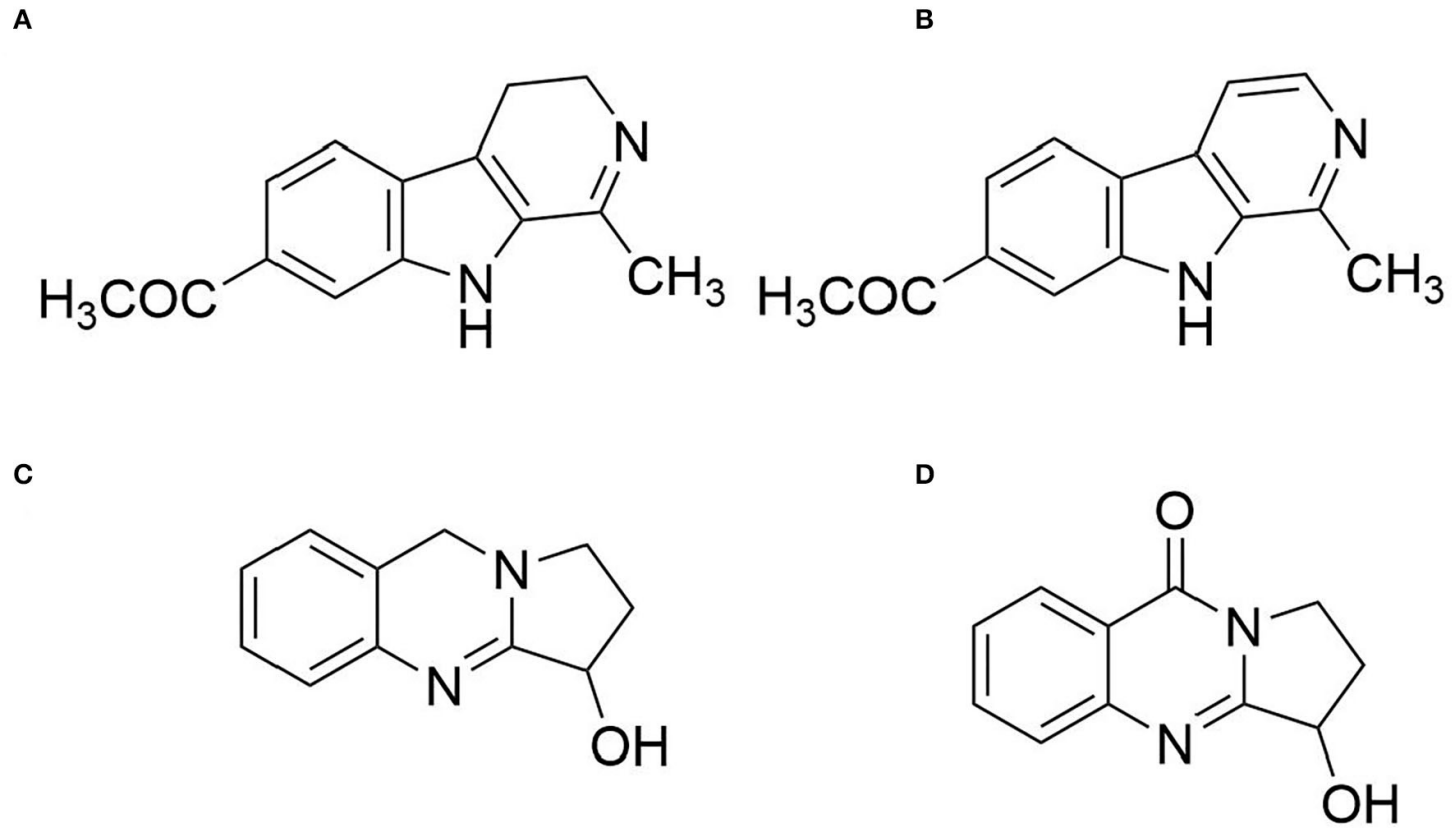
Structures of the major alkaloids found in the seeds of *P. harmala*. **(A)** Compound 1 (harmine); **(B)** Compound 2 (harmaline); **(C)** Compound 3 (vasicine); and **(D)** Compound 4 (vasicinone).

Compound **4** was obtained as a white amorphous powder. ^1^H-NMR (400 MHz DMSO-*d*_6_) δppm: 7.78 (1H, dd, *J* = 8.2, 1.1 Hz), 7.84 (1H, m), 7.53 (1H, m), 8. 16 (1H, dd, *J* = 8.1, 1.5 Hz), 4.99 (1H, t, *J* = 4.0 Hz), 4.11 (1H, m), 3.89 (1H, m), and 1.98 (2H, m); ^13^C-NMR (100 MHz DMSO-*d*_6_) δppm: 159.9 (C-2), 160.6 (C-4), 134.2 (C-5), 120.6 (C-5'), 125.8 (C-6), 127.0 (C-7), 126.35 (C-8), 149.1 (C-8'), 71.3 (C-9), 29.6 (C-10), and 43.0 (C-11). Therefore, the structure of compound **4** was determined as vasicinone by comparing the NMR data with the literature (Wang et al., 2015). Their structures are shown in Figure 1.

### HPLC Method Validation

#### Precision

The sample (No. S1) was injected six times according to the chromatographic conditions mentioned in the section HPLC analysis. The RSD values of the relative retention time of vasicine, vasicinone, harmaline, and harmine were 0.26, 0.13, 0.33, and 0.35%, respectively; the RSD of relative peak area were 1.12, 1.34, 0.82, and 0.86%, respectively.

#### Stability

The sample (No. S1) was analyzed by the aforementioned chromatography conditions after 0, 2, 4, 6, 12, and 24 h of storage. The RSD value of the relative retention time of vasicine, vasicinone, harmaline, and harmine were 0.33, 0.16, 0.33, and 0.35%, respectively; The RSD values of the relative peak area were 1.03, 2.13, 0.90, and 1.02%, respectively.

#### Repeatability

The sample (No. S1) was extracted five times in parallel by the method mentioned in Section Preparation of Samples Solutions and measured by chromatography in the aforementioned Section HPLC Analysis. The RSD values of relative retention time of vasicine, vasicinone, harmaline, and harmine were 0.22, 0.09, 0.15, and 0.15%, respectively; the RSD of relative peak area were 0.15, 0.50, 0.22, and 0.30%, respectively.

#### Linear Relationship

According to the chromatographic conditions mentioned under Section HPLC Analysis, the different mass concentrations were the transverse coordinate (X) and the peak area as the vertical ordinate (Y), and the results are shown in Table 1.

**Table 1:** Linear regression equations, linear range, and correlation coefficients for the four alkaloids.

**Components**	**Equation of linear regression**	**The linear range (μg·L^**−1**^)**	***R* ^2^**
Harmine	y = 14.757x + 260.16	60.4–3330.0	0.9998
Harmaline	y = 25.23x + 643.43	22.9–2950.0	0.9990
Vasicine	y = 18.952x + 1.4676	1.2–235.3	0.9992
Vasicinone	y = 20.104x – 14.036	0.5–102.7	0.9992

### Results of the HPLC Analysis

**Figure 2:**
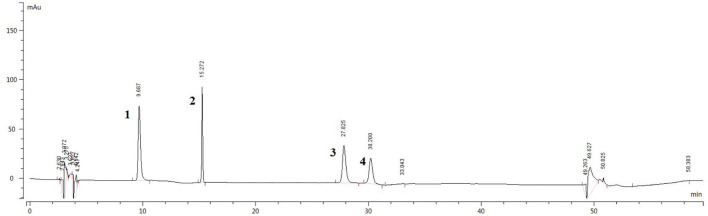
HPLC chromatogram of the standard compounds. 1. Vasicine, 2. Vasicinone, 3. Harmaline, and 4. Harmine.

**Figure 3:**
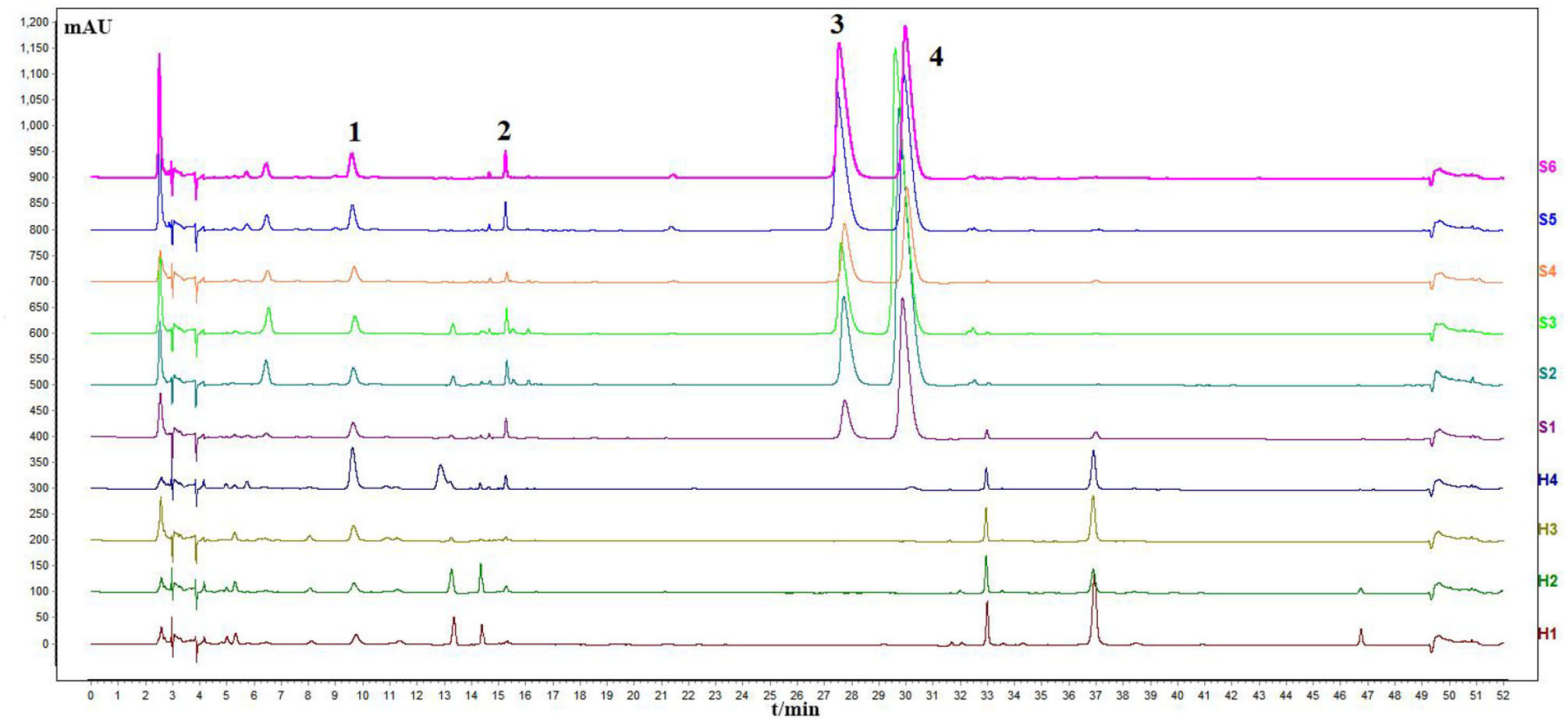
Chromatogram of HPLC content determination of *P. harmala* L. S1–S6, the seeds; H1–H4, the aerial parts.

**Figure 4:**
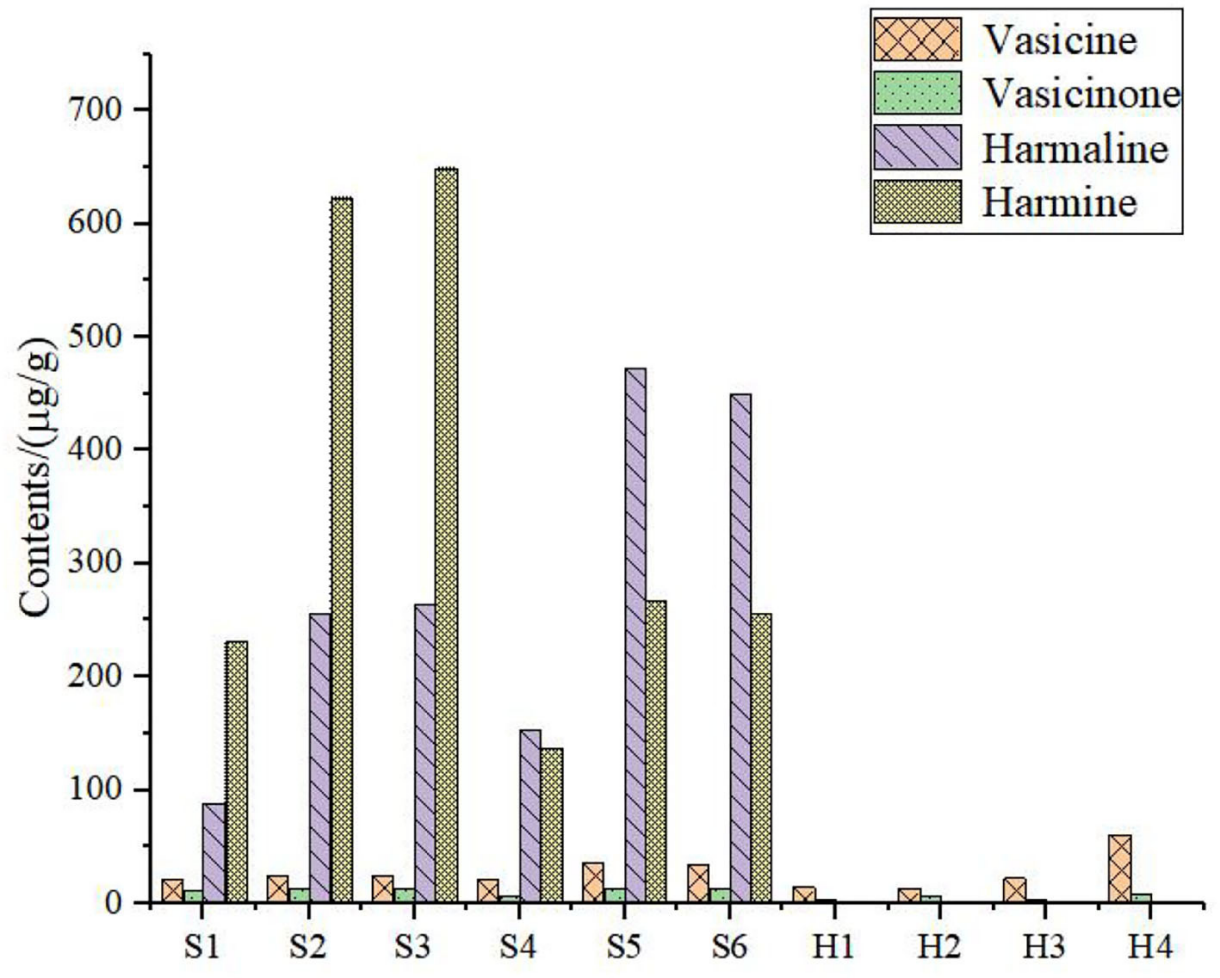
Contents of four alkaloids in *P. harmala* L. S1–S6, the seeds; H1–H4, the aerial parts.

The sample solution was prepared according to Section HPLC Chromatographic Conditions and analyzed according to the chromatographic conditions of Section HPLC Chromatographic Conditions. The chromatogram of the standard compounds is shown in Figure 2 and the HPLC chromatogram of batch 10 samples is shown in Figure 3. The contents of each index component were calculated, and the results are shown in Figure 4. The contents of vasicine, vasicinone, harmaline, and harmine in seeds were 21.36–35.27 μg·g^−1^, 5.18–13.66 μg·g^−1^, 88.05–472.23 μg·g^−1^, and 136.41–650.12 μg·g^−1^, respectively. The contents of vasicine and vasicinone in the aerial parts were 12.99–60.46 μg·g^−1^ and 2.15–8.18 μg·g^−1^, respectively, and the harmaline and harmine were not detected in the aerial parts.

### Results of the HS-SPME-GC-MS Analysis

**Figure 5:**
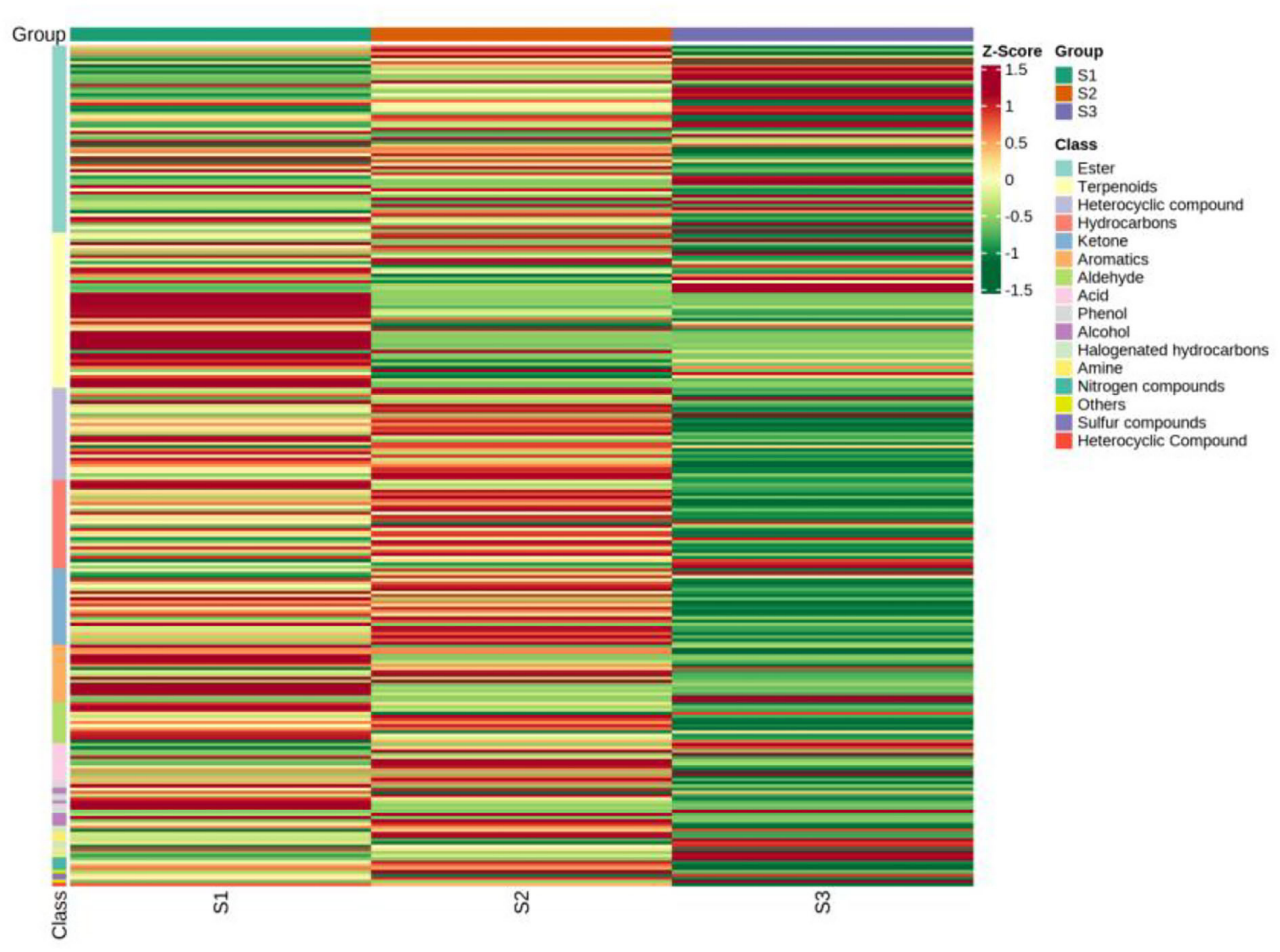
Clustering heat map of all metabolites by classification and content by GC-MS. S1, the whole herbs in Pakistan; S2, the whole herbs in Lanzhou; S3, the seeds in Xinjiang.

Based on the NIST database, the metabolites of the samples were qualitatively quantified by mass spectrometry, and the compounds with a matching degree above 80 points were selected. The mass spectrometry files were opened with MassHunter quantitative software for the integration and correction of chromatographic peaks. A total of 94 VOCs were detected by HS-SPME-GC-MS, and the metabolite numbers, values, and corresponding metabolites names of some metabolites detected in this experiment are shown in Table 2. These consisted of five alcohols, eight aromatic hydrocarbons, seven aldehydes, five acids, 17 terpenoids, 12 hydrocarbons, and 24 esters. The clustering heat map of all metabolites by classification and content is shown in Figure 5. The main volatile components in the whole herbs from Pakistan were 1,2-Benzenedicarboxylic acid, bis(2-methylpropyl) ester, Hexadecanoic acid, methyl ester, methyl salicylate, 3-methyl-4-isopropyl phenol, and propofol. The main volatile components in the whole herbs from Gansu were bis (2-methylpropyl) 1, 2-phthalate, methyl hexadecate, (Z, Z)-1,8,11, 14-heptadiene, p-xylene, ethyl hexadecanoate, butyl heptadiate-4-butyl phthalate, methyl tetradecanoate, and propofol. The main volatile components in the seed samples from Xinjiang were methyl hexadecanoate, p-xylene, octane, (Z)-9-octadecanoate, ethylbenzene, methyl octadecanoate, ethyl hexadecanoate, and methyl tetradecanoate (Table 2). Four alkaloids, caffeine, pentyl furan, isoquinoline, and 2-ethyl-furan were also detected by GC-MS.

**Table 2:** Information of volatile chemical constituents of different samples of *P. harmala* L.

**No**.	**Formula**	**RT (min)**	**Compound**	**Classify**	**CAS**	**Relative peak area**		
						**S1**	**S2**	**S3**
1	C_6_H_8_O	3.467	2-Ethyl-furan	Heterocyclic compound	3208-16-0	9.00E+00	7.58E+05	9.00E+00
2	C_5_H_12_O	4.140	1-pentol	Alcohols	71-41-0	3.69E+04	4.89E+04	2.01E+04
3	C_7_H_12_O	4.140	2,2,3-trimethylcyclobutanone	Ketone	1449-49-6	4.32E+04	4.53E+04	2.90E+04
4	C_8_H_18_	5.450	Octane	Alkanes	111-65-9	5.23E+04	1.40E+05	4.78E+06
5	C_6_H_12_O	6.687	(E)−3-hexadienol	Alcohols	928-97-2	3.35E+04	6.40E+05	3.30E+04
6	C_8_H_10_	6.765	Acetyrene	Aromatic hydrocarbon	100-41-4	2.71E+05	3.94E+05	4.04E+06
7	C_8_O_10_	6.860	Anti-dixene isotopes	Aromatic hydrocarbon	41051-88-1	6.80E+05	7.18E+05	4.67E+05
8	C_8_H_10_	6.972	p-xylene	Aromatic hydrocarbon	106-42-3	1.18E+06	2.12E+06	1.52E+07
9	C_7_H_14_O	7.654	Enanthal	Aldehyde	111-71-7	2.42E+04	2.07E+04	1.90E+04
10	C_10_H_14_	8.723	4-Methylene-1- (1-methylethyl) -biring [3.1.0] self-2-ene	Terpene	36262-09-6	6.38E+05	2.39E+04	4.04E+04
11	C_7_H_6_O	8.877	Artificial almond oil	Aldehyde	100-52-7	1.76E+05	2.13E+05	2.19E+05
12	C_10_H_16_	9.184	β-phellandrene	Terpene	555-10-2	3.21E+04	9.00E+00	3.14E+04
13	C_9_H_14_O	9.394	The pentofuran	Heterocyclic compound	3777-69-3	9.52E+04	1.59E+05	1.38E+05
14	C_10_H_14_	10.052	1-Methyl-3- (1-methylethyl) -benzene	Aromatic hydrocarbon	535-77-3	4.61E+05	4.46E+04	7.91E+04
15	C_10_H_16_	10.141	(+)-cajuputene	Terpene	5989-27-5	5.41E+04	1.04E+04	1.13E+05
16	C_8_H_8_O	10.400	Phenylacetaldehyde	Aldehyde	122-78-1	4.53E+05	2.95E+05	1.66E+05
17	C_10_H_16_	11.191	2,6-dimethyl-2,4,6-octotriene	Terpene	673-84-7	5.47E+04	1.49E+04	3.83E+03
18	C_10_H_12_	11.277	1-methyl-3- (1-methyl vinyl) -benzene	Aromatic hydrocarbon	1124-20-5	1.61E+05	3.14E+04	8.87E+03
19	C_9_H_18_O	11.510	n-nonaldehyde	Aldehyde	124-19-6	4.93E+04	7.43E+04	2.38E+04
20	C_8_H_16_O	11.646	2,6-Dimethyl-cyclohexanol	Alcohols	5337-72-4	1.93E+04	1.08E+05	4.34E+03
21	C_8_H_10_O	11.702	2-Phenyl ethanol	Alcohols	1960/12/8	8.31E+04	5.77E+04	5.71E+04
22	C_9_H_18_O_2_	11.846	Methyl caprylate	Ester	111-11-5	2.11E+04	8.04E+04	7.71E+04
23	C_10_H_16_	11.952	(E, Z)−2,6-dimethyl-2,4,6-octotriene	Alkanes	7216-56-0	4.96E+04	1.15E+05	1.44E+05
24	C_10_H_16_O	12.261	Trans-rosin phenols	Terpene	5947-36-4	9.60E+04	1.48E+03	9.00E+00
25	C_10_H_16_O	12.371	(+)-camphor	Terpene	464-49-3	3.13E+05	1.50E+04	4.98E+03
26	C_10_H_16_O	12.800	Right-menthol 1 (7), 2-dienol	Terpene	65293-09-6	6.11E+04	9.00E+00	9.00E+00
27	C_10_H_20_O	12.922	Mentha camphor	Terpene	2216-51-5	7.39E+04	1.72E+05	9.86E+03
28	C_10_H_18_O	12.988	(R)−4-methyl-1- (1-methylethyl)−3-cyclohexene-1-alcohol	Terpene	20126-76-5	2.62E+05	2.35E+04	3.83E+03
29	C_10_H_8_	13.098	Naphthalene	Aromatic hydrocarbon	91-20-3	1.58E+06	1.08E+05	2.27E+04
30	C_8_H_8_O_3_	13.193	Menthyl salicylate	Ester	119-36-8	3.00E+06	6.73E+05	1.58E+04
31	C_10_H_20_O	13.403	Capraldehyde	Aldehyde	112-31-2	1.56E+04	2.42E+04	1.87E+03
32	C_10_H_14_O	13.491	The whip said ketone	Terpene	80-57-9	2.21E+05	6.35E+03	1.27E+03
33	C_7_H_7_NO	13.666	N-phenyl, -formamide	Aromatic hydrocarbon	103-70-8	4.21E+05	1.22E+05	9.42E+03
34	C_7_H_9_NO_2_	13.857	3-Ethyl-4-methyl-1H-pyrrol-2,5-diketone	Ketone	20189-42-8	5.66E+04	1.86E+05	4.28E+03
35	C_9_H_7_N	14.084	Isoquinoline	Heterocyclic compound	119-65-3	3.78E+05	6.50E+05	1.68E+04
36	C10H12O	14.141	1- (4-ethylphenyl) -ethylketone	Ketone	937-30-4	1.49E+05	1.79E+03	9.00E+00
37	C_9_H_18_O_2_	14.472	n-nonanoic acid	Acid	112-05-0	5.77E+04	5.64E+04	9.93E+03
38	C_13_H_28_	14.685	3,8-Dimethyl-dodecane	Alkanes	17301-30-3	1.35E+04	2.87E+04	1.39E+04
39	C_10_H_14_O	14.954	3-Methyl-4-isopropylphenol	Phenol	3228/2/2	2.71E+06	1.34E+05	9.26E+03
40	C_8_H_7_N	15.062	Interaminophen acetylene	Amine	54060-30-9	4.56E+04	3.60E+04	1.86E+04
41	C_10_H_14_O	15.122	2-Methyl-5- (1-methylethyl) -phenol	Phenol	499-75-2	1.56E+06	1.74E+05	4.63E+03
42	C_13_H_28_	15.155	Tridecane	Alkanes	629-50-5	4.20E+04	6.10E+04	8.69E+04
43	C_9_H_10_O_2_	15.355	1- (2-hydroxyl-5-methphenyl) -acetone	Ketone	1450-72-2	2.60E+05	3.15E+05	1.10E+05
44	C_11_H_22_O_2_	15.550	Methyl caprate	Ester	110-42-9	4.34E+04	4.42E+04	8.18E+04
45	C_12_H_18_O	16.193	Diprivan	Phenol	2078-54-8	2.19E+06	1.37E+06	4.41E+04
46	C_12_H_25_Br	16.439	2-Brododecane	Halogenated hydrocarbon	13187-99-0	1.27E+04	1.77E+04	1.95E+04
47	C_15_H_24_	16.539	Admidia-butene	Terpene	30021-74-0	7.88E+03	1.83E+04	2.23E+05
48	C_14_H_30_	16.974	Tetradecane	Alkanes	629-59-4	1.14E+05	1.68E+05	1.24E+05
49	C_12_H_24_O	17.131	Lauraldehyde	Aldehyde	112-54-9	1.79E+04	1.14E+04	2.12E+04
50	C_13_H_22_O	17.798	Cis-leaf yl acetone	Ketone	3879-26-3	3.51E+05	6.94E+05	2.85E+04
51	C_15_H_24_	17.901	β-farnesene	Terpene	18794-84-8	6.37E+04	4.01E+04	5.17E+04
52	C_16_H_34_	18.040	2,6,10-trimethyldodecane	Alkanes	3891-99-4	2.19E+04	1.75E+05	7.40E+03
53	C_15_H_22_	18.438	α-curcumin	Terpene	644-30-4	1.87E+06	6.39E+04	1.29E+04
54	C_13_H_28_O	18.597	Tridecyl alcohol	Alcohols	112-70-9	2.23E+04	3.52E+04	1.04E+04
55	C_15_H_24_	18.663	(-)-α-Cyperylene	Terpene	469-61-4	7.64E+04	1.19E+04	7.59E+03
56	C_15_H_32_	18.732	Pentadecane	Alkanes	629-62-9	5.45E+04	6.96E+04	4.31E+04
57	C_15_H_24_O	18.796	Butylated hydroxytoluene	Aromatic hydrocarbon	128-37-0	3.00E+05	9.60E+04	1.32E+05
58	C_13_H_26_O_2_	19.102	Methyl laurate	Ester	111-82-0	2.99E+05	4.06E+05	3.77E+05
59	C_16_H_34_	19.930	3-Methylpentane	Alkanes	2882-96-4	4.12E+04	4.57E+04	2.82E+04
60	C_12_H_14_O_4_	20.190	Diethyl phthalate	Ester	84-66-2	1.46E+05	3.19E+04	3.38E+03
61	C_16_H_30_O_4_	20.207	2,2,4-trimethyl-1,3-glutarol diisobutyrate	Ester	6846-50-0	1.13E+05	6.90E+04	1.03E+04
62	C_14_H_28_O_2_	20.282	Ethyl laurate	Ester	106-33-2	4.92E+04	8.49E+04	1.95E+04
63	C_16_H_34_	20.416	Hexadecane	Alkanes	544-76-3	1.57E+05	1.51E+05	9.56E+04
64	C_15_H_18_O_2_	20.469	I cerofuran ketone	Terpene	20493-56-5	3.46E+05	5.65E+03	9.00E+00
65	C_15_H_26_O	20.703	Deodar	Terpene	77-53-2	6.45E+04	1.19E+05	7.49E+03
66	C_19_H_33_F_5_O_2_	21.317	Pentafluoropropionlic acid, 4-hexadyl ester	Ester	1000283-04-1	2.34E+04	2.70E+04	1.16E+04
67	C_12_H_12_O_2_	21.546	Z-butadiene	Alkanes	72917-31-8	1.10E+05	4.99E+04	9.00E+00
68	C_17_H_36_	21.841	Dioctylmethane	Alkanes	629-78-7	1.22E+05	5.54E+04	2.82E+04
69	C_15_H_22_O_2_	21.942	Benzoic acid 2-ethylhexanide	Ester	5444-75-7	5.26E+04	5.05E+04	4.23E+03
70	C_15_H_30_O	22.000	Is the glutenal	Aldehyde	2765/11/9	8.03E+05	7.21E+04	7.38E+03
71	C_15_H_30_O_2_	22.083	Methyl myristate	Ester	124-10-7	3.42E+05	1.44E+06	1.77E+06
72	C_14_H_30_O_3_S	22.130	Nrenyl ulfite	Ester	1000309-14-2	2.47E+04	4.09E+04	9.00E+00
73	C_12_H_14_O_2_	22.205	Artemicen lactone	Ester	81944-09-4	6.20E+04	2.43E+04	9.00E+00
74	C_15_H_22_O	22.330	(R)−5- (1,5-dimethyl-4-hexenenyl)−2-methyl-phenol	Phenol	30199-26-9	3.24E+06	6.01E+04	4.76E+03
75	C_14_H_28_O_2_	22.419	Texanic acid	Acid	544-63-8	1.27E+04	4.82E+05	3.29E+04
76	C_16_H_32_O_2_	22.712	Ethyl tetraheatanate	Ester	124-06-1	5.34E+04	2.93E+05	8.77E+04
77	C_18_H_38_	22.783	2,6,10-trimethyl-pentane	Alkanes	3892-00-0	8.21E+04	5.45E+04	3.42E+04
78	C_15_H_28_O_2_	22.837	2-Hydroxycyclohexanone	Ketone	4727-18-8	3.21E+04	6.22E+04	4.84E+04
79	C_16_H_32_O_2_	22.962	Methyl sevenate	Ester	7132-64-1	1.57E+05	1.34E+05	3.26E+05
80	C_8_H_10_N_4_O_2_	23.133	Caffeine	Heterocyclic compound	1958/8/2	4.59E+05	5.45E+05	3.99E+05
81	C_16_H_22_O_4_	23.232	1,2-phenylic acid double (2-methylpropyl) ester	Ester	84-69-5	1.19E+07	1.43E+07	8.53E+05
82	C_20_H_40_O	23.361	3,7,11,15-Tetraethyl-2-dohexadecene-1-alcohol	Terpene	102608-53-7	1.47E+05	2.26E+05	1.38E+04
83	C_18_H_32_O_2_	23.432	Linoleic acid	Acid	506-21-8	9.78E+05	4.12E+05	1.14E+05
84	C_17_H_32_O_2_	23.516	(Z)−9-methyl dohexaenoacrylate	Ester	1120-25-8	6.90E+04	2.68E+05	8.00E+05
85	C_17_H_34_O_2_	23.688	Methyl palmitate	Ester	112-39-0	4.02E+06	9.42E+06	2.61E+07
86	C_19_H_28_O_4_	23.845	Butane-4-butylester phthalate	Ester	1000356-78-4	1.28E+06	1.66E+06	8.64E+04
87	C_18_H_36_O_2_	24.035	Acetate hexadecanate	Ester	628-97-7	5.53E+05	2.00E+06	2.89E+06
88	C_19_H_36_O_2_	24.084	(Z) -Ethyl hexadienylate	Ester	1089325-29-0	4.85E+04	6.77E+04	1.20E+05
89	C_18_H_36_O_2_	24.203	Xamyl hexaecate	Ester	6929-04-0	7.97E+04	1.86E+05	3.10E+05
90	C_19_H_36_O_2_	24.659	(Z)−9-methyl octacaracene	Ester	112-62-9	3.87E+05	4.67E+05	4.52E+06
91	C_19_H_38_O_2_	24.726	Methyl octaenate	Ester	112-61-8	3.26E+05	6.01E+05	3.29E+06
92	C_18_H_34_O_2_	24.781	Cis oxknee acid	Acid	506-17-2	9.00E+00	5.09E+05	9.00E+00
93	C_20_H_38_O_2_	25.439	Cis-11-eicosaric acid	Acid	5561-99-9	7.32E+03	1.46E+04	1.92E+05
94	C_19_H_34_O_2_	26.159	(Z, Z) 9,12-18 endocardienylate-methyl ester	Ester	112-63-0	9.00E+00	5.52E+03	1.16E+04

### Antimicrobial Activity

#### Results of Antibacterial Activity

The petroleum ether extract and methanol extract of *P. harmala* showed broad-spectrum antibacterial activity against all the tested bacteria, as shown in Table 3. At the concentration of 800 μg·ml^−1^, the methanol extract of seeds displayed the highest bioactivity against *E. coli* ATCC 25922 with an inhibition rate of more than 90% and the petroleum ether extract of seeds showed slight inhibition against *Staphylococcus aureus*. The methanol and petroleum ether extracts of the aerial parts were only slightly against *Candida albicans* ATCC 24433 with inhibition rates of 20.08 and 15.07%, respectively.

**Table 3:** Antibacterial activity of extracts and alkaloids from *P. harmala* L.

**Sample**	**Concentration (μg·mL^**−1**^)**	**Average inhibition rate** **±SD (%) (*****n*** **=** **3)**				
		***E. coli***	***S. aureus***	***X. oryzae***	***P. atroseptica***	***X. axonopodis***
E1	800	99.46 ± 0.11**	76.95 ± 3.38**	92.61 ± 0.25**	47.34 ± 4.28*	100 ± 0.00**
	400	92.62 ± 2.00**	15.80 ± 3.25	88.14 ± 2.29**	–	97.74 ± 0.97**
	200	21.24 ± 2.47	–	64.07 ± 1.21**	–	89.68 ± 1.81**
	100	5.50 ± 2.58	–	37.56 ± 3.62	–	67.42 ± 0.00**
	50	–	–	15.84 ± 2.90	–	67.06 ± 0.97**
E2	800	–	46.68 ± 1.07*	48.05 ± 4.33*	32.15 ± 1.58	27.86 ± 1.47
E3	800	–	–	59.51 ± 4.84*	31.25 ± 8.21	–
E4	800	–	–	68.68 ± 3.57*	24.49 ± 2.81	–
C1	100	50.11 ± 2.50*	13.92 ± 0.98	2.65 ± 0.88	27.44 ± 0.61	–
	50	32.01 ± 1.18	7.14 ± 1.10	2.65 ± 1.77	3.66 ± 1.83	–
C2	100	90.68 ± 1.18**	12.64 ± 1.47	0.44 ± 0.44	77.74 ± 2.13**	–
	50	61.37 ± 1.47**	9.16 ± 0.61	–	40.55 ± 1.52*	–
	25	38.59 ± 0.86*	–	–	–	–
	12.5	9.93 ± 1.33	–	–	–	–
C3	100	32.26 ± 0.18*	15.32 ± 2.56	–	19.50 ± 0.00	–
	50	12.34 ± 1.32	7.51 ± 1.59	–	–	–
C4	100	25.34 ± 0.37	–	1.33 ± 0.44	22.13 ± 0.58	–
	50	5.29 ± 1.25	–	–	–	–

E1, The methanol extract of seed; E2, The petroleum ether extract of seed; E3, The methanol extract of the aerial parts; E4, The petroleum ether extract of the aerial parts; C1, Harmine; C2, Harmaline; C3, Vasicine; C4, Vasicinone. *P < 0.05, **P < 0.01 compared with the control group.

The methanol extract of seeds displayed the highest bioactivity against *X. oryzae*, and the inhibition rate was 92.61% at 800 μg·ml^−1^. The petroleum ether extract of seeds and the aerial parts and the methanol extract of seeds displayed moderate bioactivity against *X. oryzae*, and the inhibition rates were from 48.05 to 68.68% at 800 μg·ml^−1^.

Citrus canker is the most serious Citrus bacterial disease caused by the gram-positive *X. axonopodis;* it can affect plant growth, fruit quality, and yield, which caused great economic losses to the citrus industry (Sun et al., 2019). In the present study, the methanol extract of seeds was effective against *X. axonopodis*, the inhibition rates were 67.42 and 100% at 50 and 800 μg·ml^−1^, respectively. The petroleum ether extract of seeds was slightly against *X. axonopodis* with an inhibition rate of 27.86% at 800 μg·ml^−1^.

As shown in Table 3, methanol extract of seeds exhibited moderate antimicrobial activity against *P. atroseptica* ACCC 19901, and the inhibition rate was 47.34% at 800 μg·ml^−1^. Petroleum ether extract of seeds and the aerial parts and methanol extract of seeds also exhibited slight antimicrobial activity against *P. atroseptica* ACCC 19901, and the inhibition rates were 32.15, 31.25, and 24.49%, respectively.

#### Antifungal Activity of *P. harmala* Extracts

The antifungal activities of *P. harmala* extract against pathogenic fungi are shown in Table 4. At the concentration of 500 μg·ml^−1^, the methanol extract of seeds effectively inhibited seven fungi of *Rhizoctonia solani, Sclerotinia sclerotiorum, Botrytis cinerea, Fusarium graminearum, Magnaporthe oryzae, Phytophthora capsici*, and *Candida albicans* ATCC 24433, with inhibition rates of 50.33, 64.32, 76.32, 52.98, 42.57, 55.58, and 47.47%, respectively. The petroleum ether extract of seeds weakly inhibited *Rhizoctonia solani* and *Magnaporthe oryzae*, with inhibition rates of 10.88 and 10.88%, respectively. The methanol and parts petroleum extract of aerial parts only weakly suppressed *Fusarium graminearum* and *Magnaporthe oryzae*, with inhibition rates of 10.89% and 10.7%, and 12.08% and 16.57%, respectively. The methanol extracts of seeds showed stronger antifungal activities with a wide antifungal spectrum. Our results showed that the aerial parts and seed extracts have broad-spectrum antibacterial activities against agricultural pathogenic bacteria, which may be related to its alkaloids (Adnan et al., 2019; Zhang et al., 2020), but its specific active components and its antibacterial mechanism need to be further studied.

**Table 4:** Antifungal activity of extracts and alkaloids from *P. harmala* L.

**Sample**	**Average inhibition rate** **±SD (%) (*****n*** **=** **3)**						
	***R. solani***	***S. sclerotiorum***	***F. graminearum***	***M. oryzae***	***P. racapsici***	***C. albicans***	***B. cinerea***
E1	50.33 ± 0.11**	64.32 ± 0.98**	52.98 ± 0.89**	42.57 ± 0.9**	55.58 ± 0.44**	47.47 ± 1.18*	76.32 ± 0.88**
E2	10.88 ± 0.16	–	–	–	13.57 ± 0.64	25.32 ± 2.16	–
E3	–	–	10.89 ± 1.21	–	10.70 ± 0.49	20.08 ± 4.33	–
E4	–	–	12.08 ± 0.34	–	16.57 ± 1.78	15.07 ± 2.65	–
C1	21.89 ± 1.33	18.67 ± 1.02	15.23 ± 0.54	21.93 ± 1.67	20.37 ± 1.56	–	–
C2	13.32 ± 2.16	23.45 ± 2.32	24.12 ± 1.48	15.23 ± 1.15	29.05 ± 2.27*	–	–
C3	16.12 ± 1.78	19.87 ± 0.59	19.26 ± 0.68	16.38 ± 2.56	13.56 ± 1.33	–	–
C4	24.36 ± 1.36	15.38 ± 1.38	13.63 ± 1.12	10.81 ± 0.26	14.80 ± 0.68	7.15 ± 0.56	–

E1, The methanol extract of seed; E2, The petroleum ether extract of seed; E3, The methanol extract of the aerial parts; E4, The petroleum ether extract of the aerial parts; C1, Harmine; C2, Harmaline; C3, Vasicine; C4, Vasicinone. *P < 0.05, **P < 0.01 compared with the control group.

Furthermore, we evaluated the antimicrobial activity of four alkaloids isolated from *P. harmala*, including *Rhizoctonia solani, Sclerotinia sclerotiorum, Magnaporthe oryzae, Fusarium graminearum*, and *Phytophthora capsici*, and found that the growth of all five strains was inhibited in the presence of alkaloids (Tables 3, 4), while harmine and harmaline showed broad-spectrum antibacterial activities against *X. oryzae* (ACCC 11602), *E. coli* ATCC 25922, and *Staphylococcus aureus* (Newman). These findings indicated that alkaloids could account for antimicrobial activity. Furthermore, harmine and harmaline showed better antibacterial activities, which could be used as lead molecules in the development of new antimicrobial drugs. In the future study, we are going to select harmine and harmaline as lead compounds, and a series of derivatives are supposed to design and synthesize to evaluate their antifungal activities and preliminary antifungal mechanism.

## Conclusion

In conclusion, the aerial parts and seeds of *Peganum harmala* L showed a large difference in the content of four alkaloids. Harmine and harmaline were mainly found in seeds, while the aerial parts mainly contained vasicine and vasicinone. A total of 94 metabolites were detected by HS-SPME-GC-MS technology for the first time. The VOCs of *P. harmala* mainly consisted of hydrocarbons, acids, ketones, aromatic hydrocarbons, esters, and heterocyclic compounds. In addition, the extracts of the aerial parts and seeds showed antibacterial activities against 12 bacterial strains and fungi. This study demonstrates that the alkaloids from *P. harmala* are a potentially non-toxic and ecofriendly botanical fungicide for the management of fungi.

## Data Availability Statement

The original contributions presented in the study are included in the article/Supplementary Material, further inquiries can be directed to the corresponding author/s.

## Author Contributions

Methodology, conceptualization, investigation, and writing—original draft preparation: NW. Supervision, writing—review and editing, and project administration: ZY. Investigation and formal analysis: JA and ZZ. Writing—review and visualization: YL and JF. All authors have read and agreed to the published version of the manuscript.

## Funding

This work was supported by the Key Project of NMPA Key Laboratory for Quality Control of Traditional Chinese Medicine (2020GSMPA-KL11), the Natural Science Foundation of Gansu Province (20JR5RA311 and 20JR5RA216), the Key Program for International S&T Cooperation Projects of Gansu Province (18YF1WA115), and the Fundamental Research Funds for the Central Universities (lzujbky-2021-kb40 and lzujbky-2020-46).

## Conflict of Interest

The authors declare that the research was conducted in the absence of any commercial or financial relationships that could be construed as a potential conflict of interest.

## Publisher's Note

All claims expressed in this article are solely those of the authors and do not necessarily represent those of their affiliated organizations, or those of the publisher, the editors and the reviewers. Any product that may be evaluated in this article, or claim that may be made by its manufacturer, is not guaranteed or endorsed by the publisher.
