# Supplementary material for: The Antimicrobial Activity and Characterization of Bioactive Compounds in Peganum harmala L. Based on HPLC and HS-SPME-GC-MS
Source: Front Microbiol. 2022 Jul 19;13:916371. doi: 10.3389/fmicb.2022.916371 (PMC9343986; doi:10.3389/fmicb.2022.916371)
Supplement: Supplementary file 1 [file Data_Sheet_1.DOCX]

**Supplementary Data**

**The Antimicrobial Activity and Characterization of Bioactive Compounds in Pegannum harmala L. based on HPLC and HS-SPME-GC-MS**

Ningning Wang^1^, Junxia An^1^, Zhijun Zhang^1,2^, Yingqian Liu^1,2^, Jianguo Fang^3^, Zhigang Yang^*1,2^

1. School of Pharmacy, Lanzhou University, Lanzhou 730000, China;
2. Collaborative Innovation Center for Northwestern Chinese Medicine, Lanzhou University, Lanzhou 730000, China;
3. School of Chemistry and Chemical Engineering, Lanzhou University, Lanzhou 730000, China;


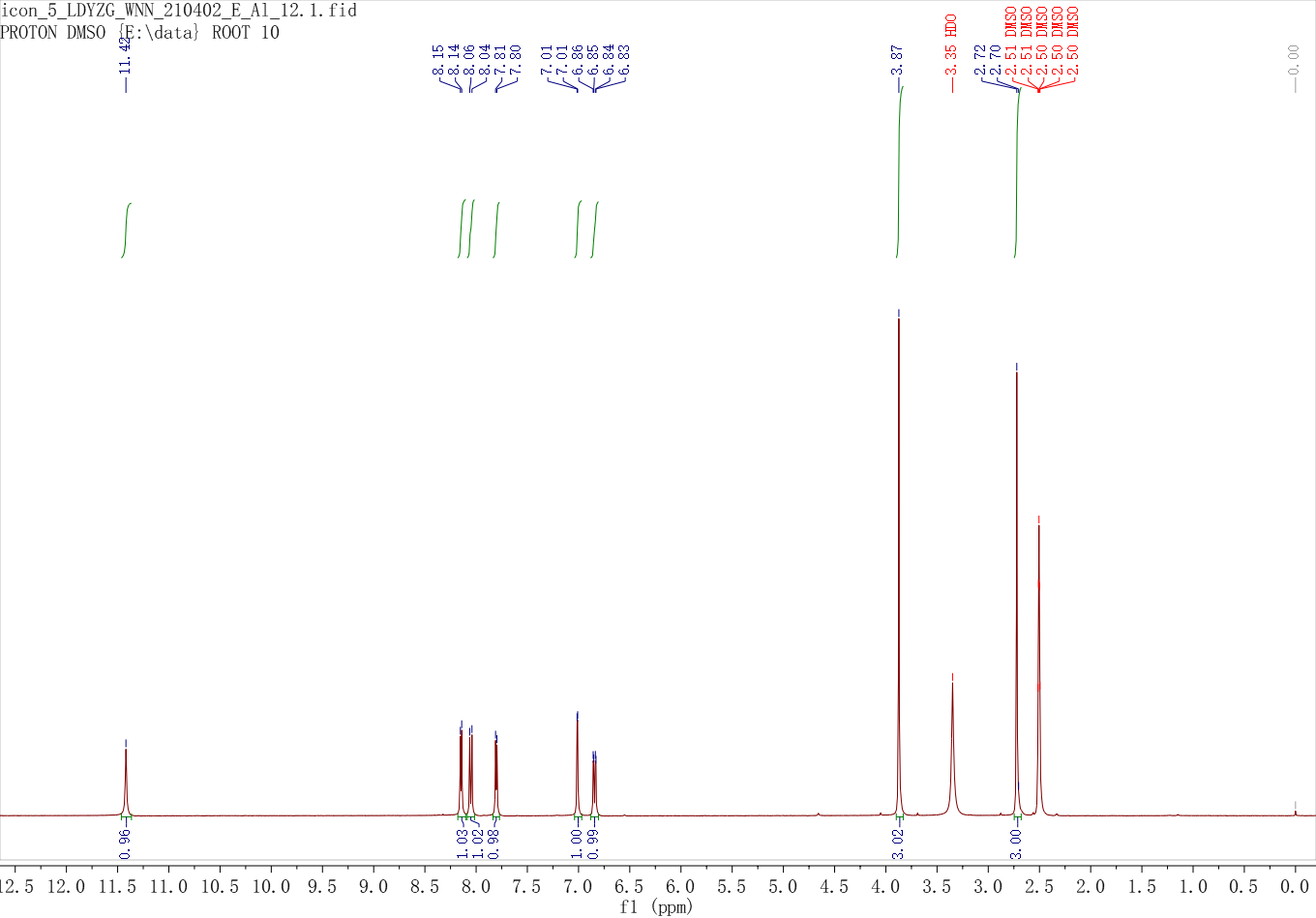


Figure S1: ^1^H-NMR of Compound 1


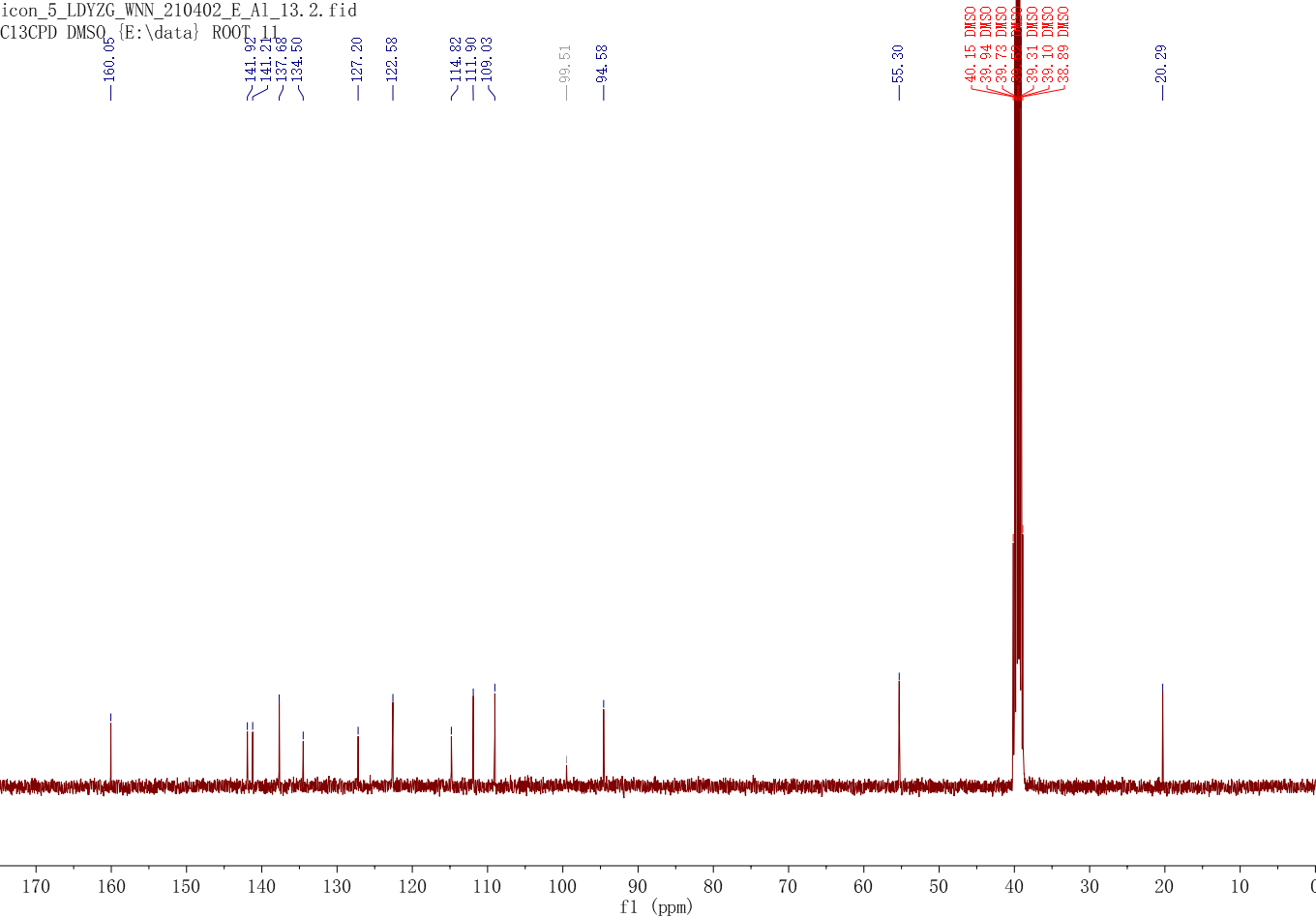


Figure S2: ^13^C-NMR of Compound 1


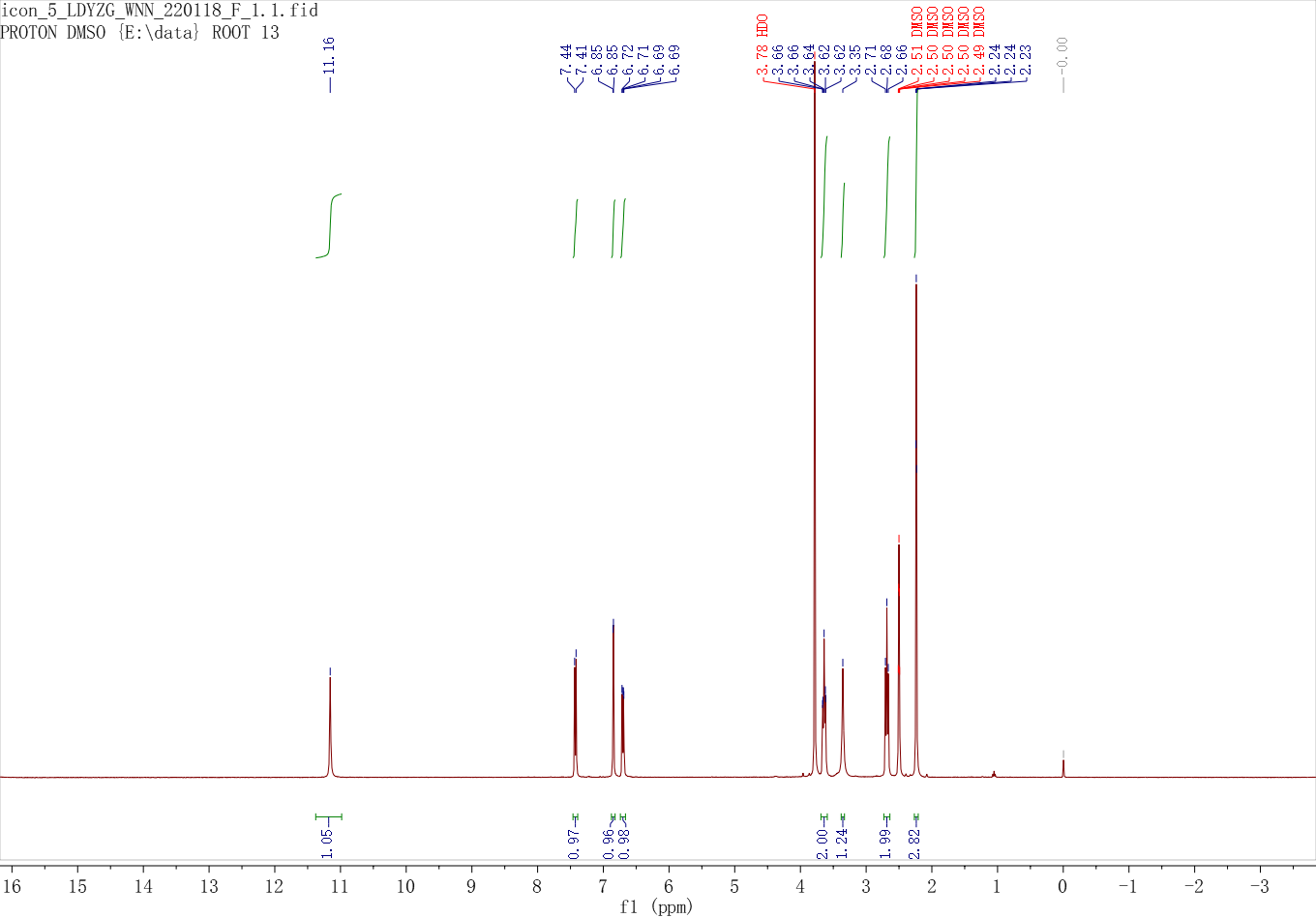


Figure S3: ^1^H-NMR of Compound 2


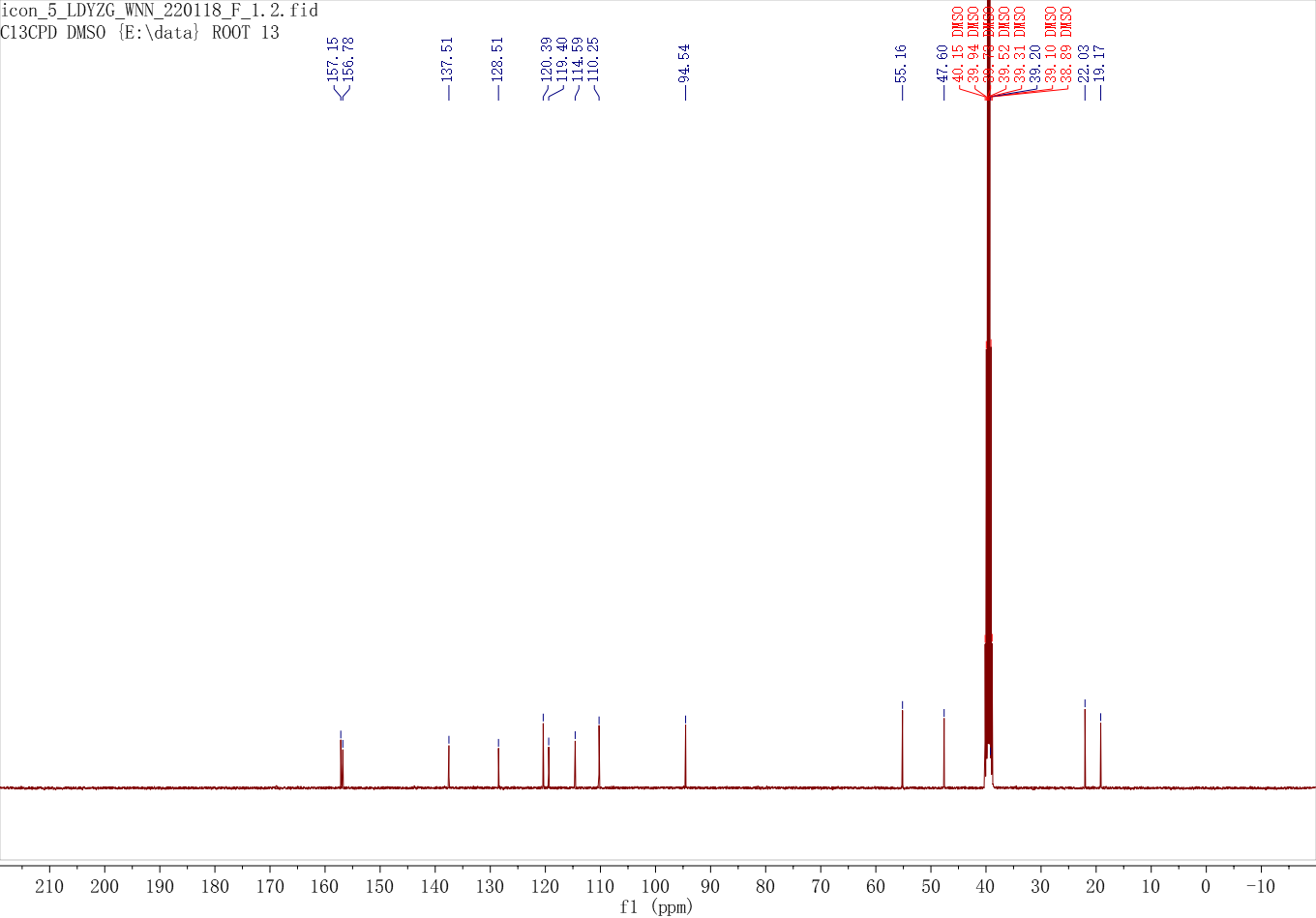


Figure S4: ^13^C-NMR of Compound 2


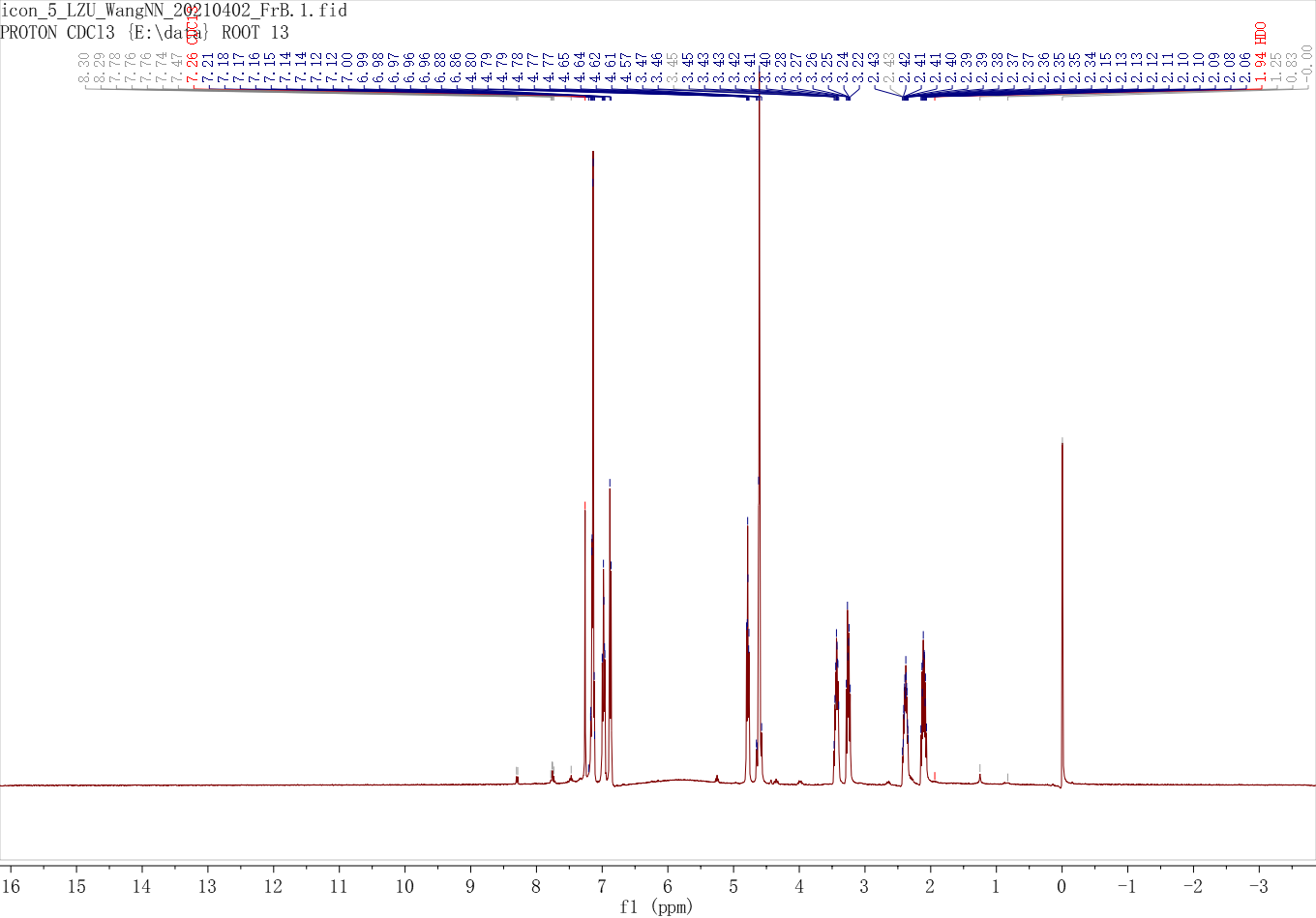


Figure S5: ^1^H-NMR of Compound 3


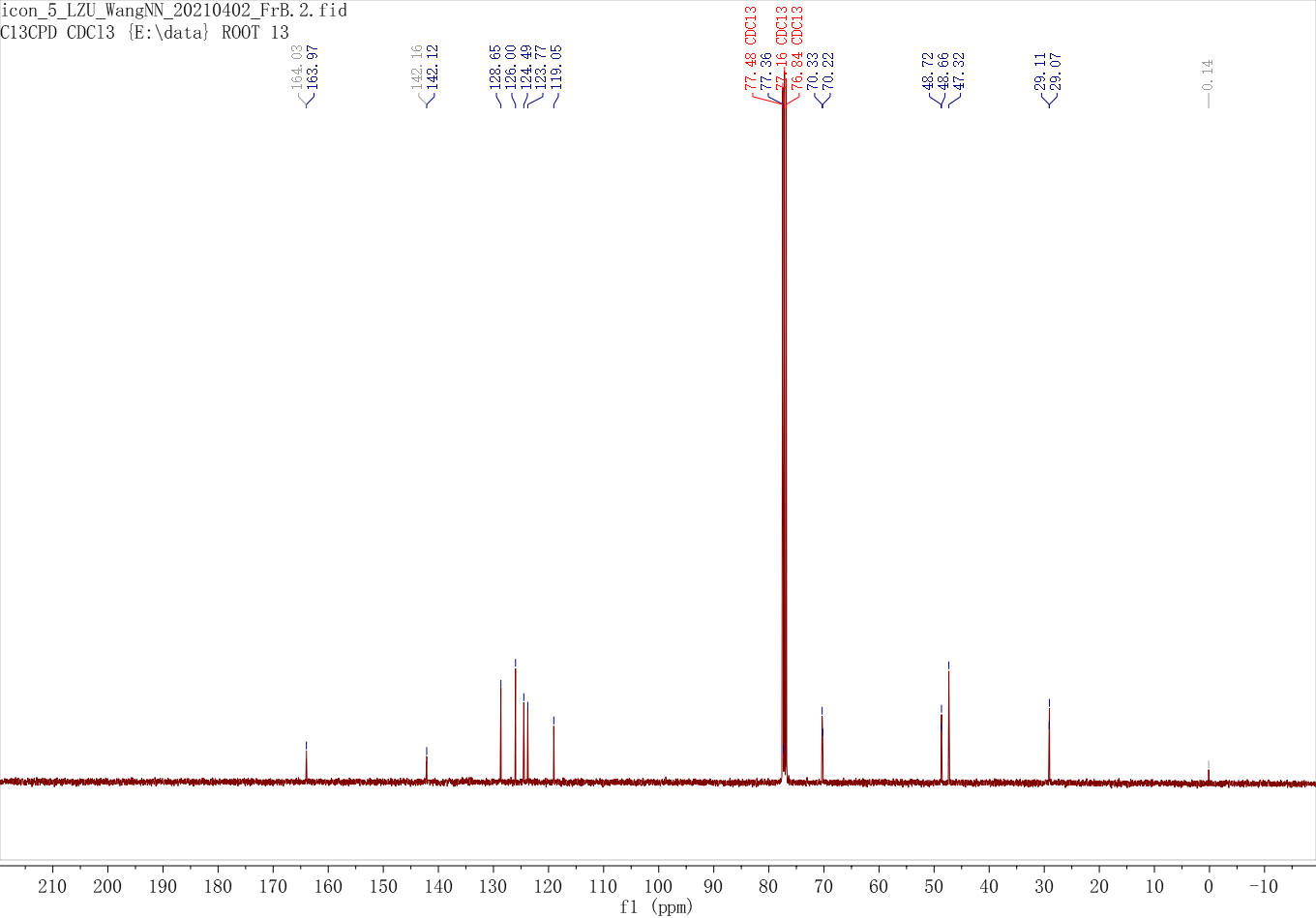


Figure S6: ^13^C-NMR of Compound 3


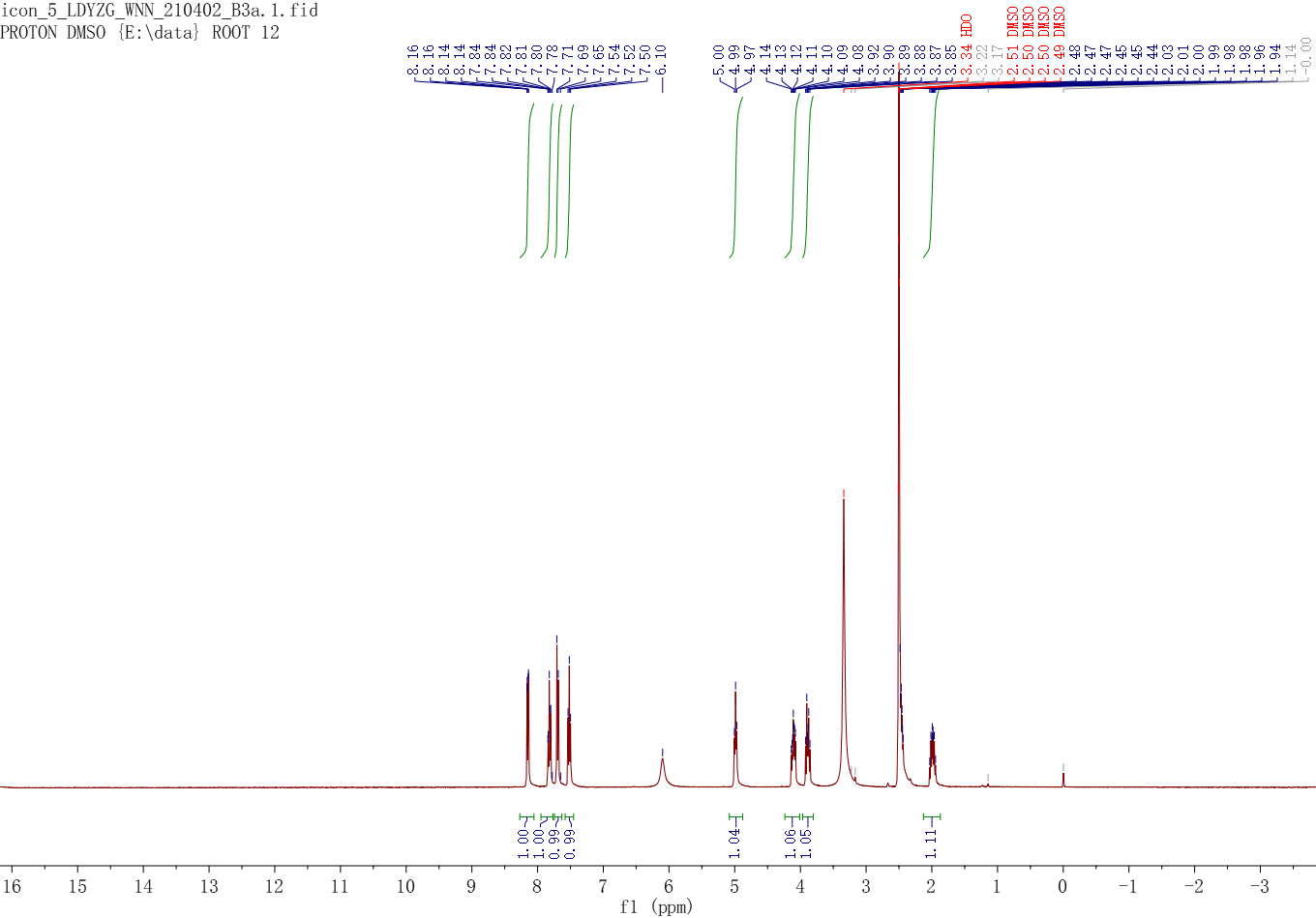


Figure S7: ^1^H-NMR of Compound 4


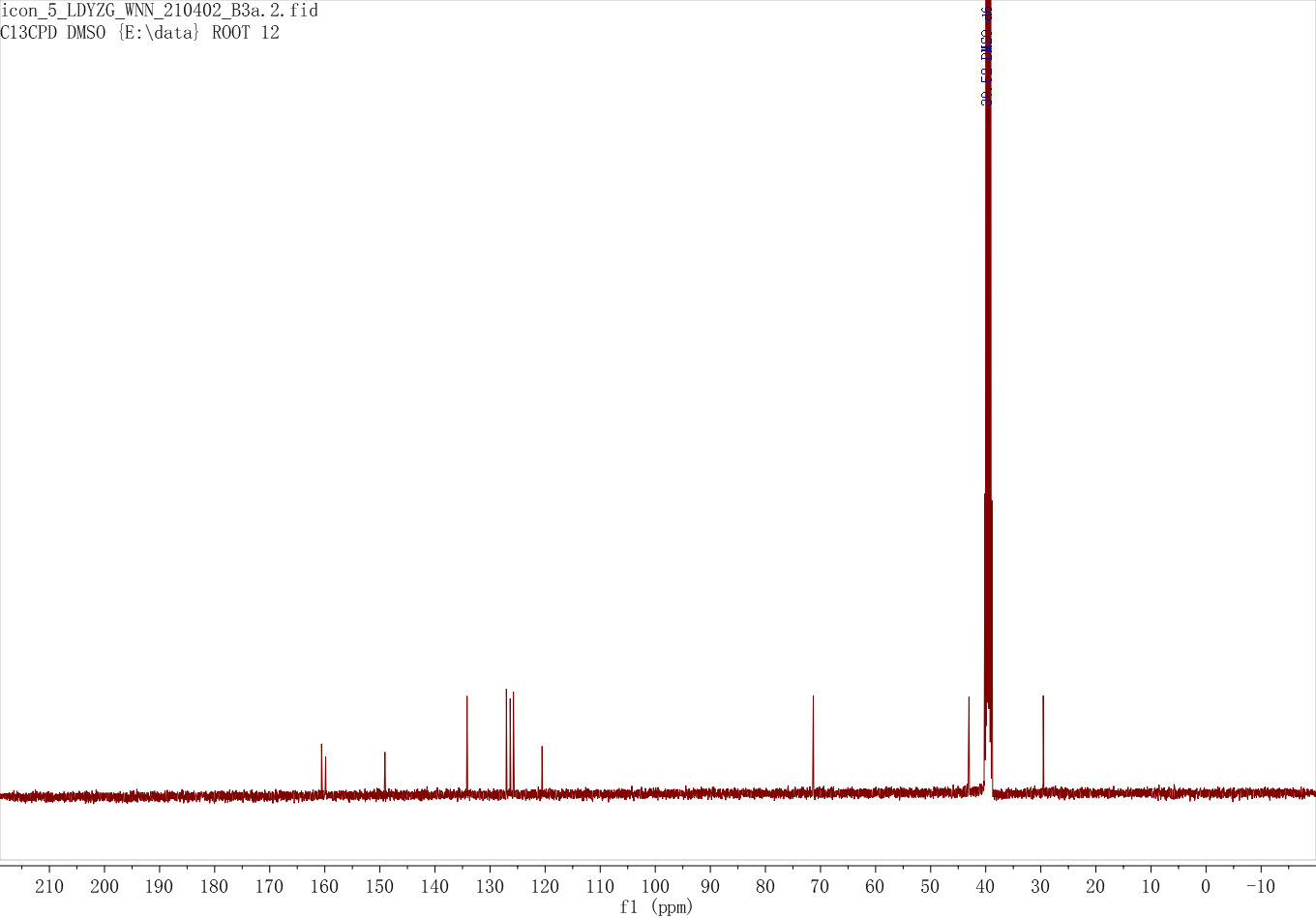


Figure S8: ^13^C-NMR of Compound 4
